# Acute Esophageal Necrosis “Black Esophagus” in a Patient With Advanced Renal Cell Carcinoma: An Autopsy Case Report

**DOI:** 10.1155/crip/7502949

**Published:** 2026-08-19

**Authors:** Kamaleldein Einar, Niki Shrestha, Dayne Ashman, Omar Samarah, Issa Snoubar, Akram Shalaby

**Affiliations:** ^1^ Department of Family Medicine, University of Arkansas for Medical Sciences, Little Rock, Arkansas, USA, uams.edu; ^2^ Department of Pathology, University Hospitals Cleveland Medical Center/Case Western Reserve University, Cleveland, Ohio, USA; ^3^ Department of Medicine, An-Najah National University, Nablus, West Bank, State of Palestine, najah.edu

## Abstract

Acute esophageal necrosis (AEN), also called “black esophagus,” is a rare, life‐threatening condition characterized by circumferential black discoloration of the esophageal mucosa, usually secondary to ischemia, impaired mucosal defenses, and gastric acid injury. It is often associated with severe debilitating illnesses, diabetes mellitus, malignancy, and hemodynamic shock, among other comorbidities. We report a female patient in her 80s with a history of breast cancer who presented with gross hematuria and was diagnosed with advanced clear cell renal cell carcinoma with inferior vena cava thrombosis. She underwent radical nephrectomy with vascular reconstruction, and the postoperative course was complicated by bleeding and sudden cardiopulmonary collapse on the second postoperative day, leading to death. Autopsy revealed acute tubular necrosis, cardiomegaly, diffuse alveolar damage, and circumferential black discoloration and necrosis of the distal esophagus, consistent with AEN. To our knowledge, this is the first case of AEN in the setting of renal cell carcinoma reported in the English language literature. The condition was not clinically suspected during hospitalization, underscoring its diagnostic challenge and potential under‐recognition.

## 1. Introduction

Acute esophageal necrosis (AEN), also known as “black esophagus,” “Gurvits syndrome”, or “acute necrotizing esophagitis,” is a rare entity characterized by diffuse and circumferential black discoloration of the distal esophagus, of variable length. The discoloration typically stops abruptly at the gastroesophageal junction (GEJ) [1]. Nevertheless, this process often extends to involve the proximal parts of the esophagus.

The prevalence of AEN ranges from 0.06% to 0.28% in upper gastrointestinal (GI) endoscopies [2, 3]. In autopsy studies, the prevalence of AEN was found to be between 0% and 0.2% [4, 5]. The true prevalence of AEN is unknown and is likely underestimated given that early cases due to transient ischemic or chemical injury could resolve spontaneously without the need for upper GI endoscopy [6].

An expanded list of risk factors has been associated with AEN, including older age, male sex, cardiovascular disease, diabetes mellitus, alcohol use disorder, hemodynamic instability, malnutrition, gastric outlet obstruction, renal insufficiency, hematologic and solid organ malignancy, and hypercoagulable state, among others [1, 7]. The majority of patients present with upper GI bleeding in the form of hematemesis, coffee ground emesis, or melena [3]. Other potential symptoms at presentation include epigastric or abdominal pain, nausea, vomiting, dysphagia, light‐headedness, or dyspnea [8]. Anemia is commonly present in blood tests. Endoscopic examination of patients with AEN characteristically reveals circumferential black discoloration of the distal esophageal mucosa with sharp demarcation at the GEJ [1].

Management of AEN is typically supportive with symptomatic relief and correction of underlying comorbidities. AEN generally carries a poor prognosis, particularly in the presence of life‐threatening complications.

In this case, we present a female patient who presented with urinary symptoms and gross hematuria and was later diagnosed with advanced renal cell carcinoma (RCC) with extended inferior vena cava (IVC) thrombosis. AEN was detected on autopsy.

## 2. Case Presentation

**Figure 1:**
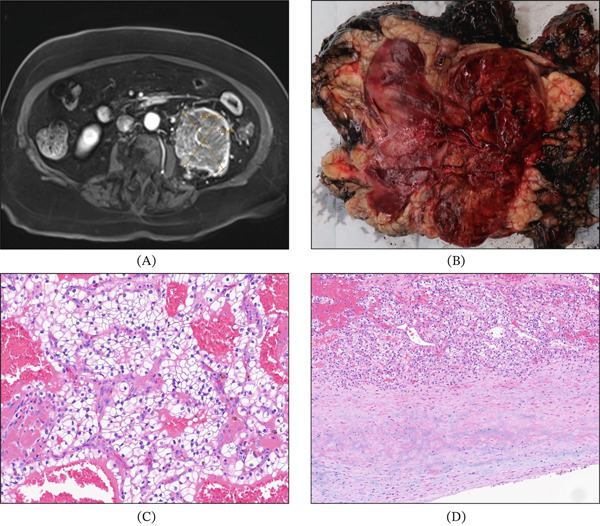
(A) Abdominal MRI shows an 8‐cm left renal mass. (B) Gross examination of the nephrectomy specimen reveals a large mass occupying the majority of the left kidney. (C) Histological section from the kidney shows clear cell renal cell carcinoma (hematoxylin–eosin, 20x magnification). (D) Clear cell renal cell carcinoma (upper half) attached to vessel wall (lower half) (hematoxylin–eosin, 4x magnification).

We present a female patient in her 80s with a past medical history of cervical dystonia, tremors, and remote breast cancer. She was diagnosed with breast cancer (ER+/PR+) 18 years prior to the presentation (pathologic stage: pT1b pN0 pM0) and underwent lumpectomy and axillary lymph node dissection followed by radiation and anastrozole (Arimidex) therapy. She presented to the emergency department at an outside institution with 2 weeks of gross hematuria, frequency, and dysuria. She denied abdominal or flank pain, chills, fever, nausea, vomiting, or shortness of breath. At the outside hospital, her hemoglobin was critically low, requiring packed red blood cells (PRBCs) transfusion. Abdominal magnetic resonance imaging (MRI) revealed an 8 × 7.2 cm left renal mass (Figure 1A) with a 9.3 × 3.2 cm thrombus extending into the left renal vein and IVC. Blood was seen in the urinary bladder with no definitive enhancing mass or urinary bladder wall thickening. Given the suspicion of RCC with a tumor thrombus, she was transferred to our institution for further management.

On admission, she remained hemodynamically stable but continued to pass clots via Foley catheter. Hemoglobin was 5.5 g/dL, creatinine 2 mg/dL, and urine cultures were negative. She received additional PRBCs transfusions and continuous bladder irrigation. Her hemoglobin incremented appropriately and became stable with transfusions. An abdominal computed tomography (CT) scan showed no evidence of metastatic disease. A bedside flexible cystoscopy showed no bladder lesions. She underwent left open radical nephrectomy with IVC thrombectomy and vascular reconstruction. The surgery lasted approximately 6 h. The patient lost an estimated 1000 mL of blood and was given 700 mL of PRBCs intraoperatively. A dose of ephedrine was given for an episode of hypotension during surgery. The nephrectomy specimen showed a 6.5 cm mass involving the middle and lower poles of the left kidney (Figure 1B). Histological examination revealed clear cell RCC, WHO/ISUP grade 3 (Figure 1C), with perinephric, renal sinus, and renal vein involvement (pathologic stage: pT3c pN0); eight lymph nodes were negative. A separate specimen from the IVC thrombus demonstrated fragments of clear cell RCC involving portions of vessel wall (Figure 1D).

The postoperative course was complicated by epistaxis and upper GI bleeding. The postoperative systolic blood pressure ranged from 100 to 141 mmHg and the diastolic blood pressure ranged from 52 to 66 mmHg. Heart rate ranged from 77 to 107 bpm and temperature was normal. Laboratory testing showed hemoglobin of 8.2 g/dL and hematocrit of 24.1. Lactate levels were normal, 1.6–1.8 mmol/L. No vasopressors were administered postoperatively. On postoperative Day 2, she was found unresponsive with dark brown nasal discharge; resuscitation was unsuccessful. The patient was pronounced dead, and an autopsy was authorized by the legal next of kin.

**Figure 2:**
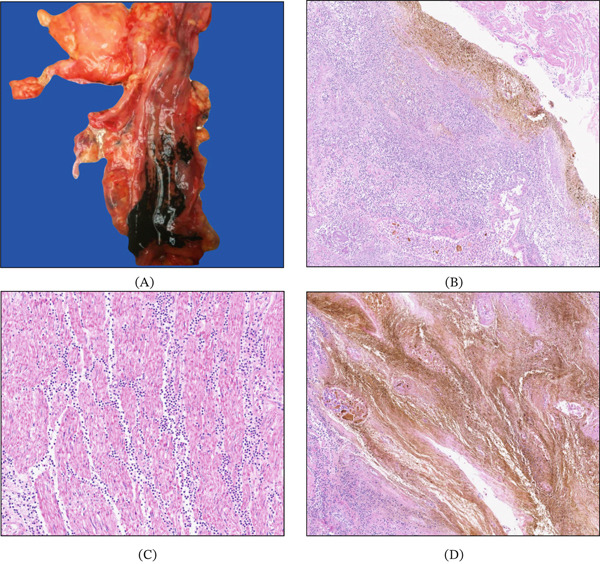
(A) Gross image of the esophagus shows black discoloration of the distal esophageal mucosa that abruptly ends at the gastroesophageal junction. Histological sections from the esophagus demonstrate (B) esophageal mucosal necrosis (hematoxylin–eosin, 4x magnification), associated with (C) transmural neutrophilic infiltrate (hematoxylin–eosin, 10x magnification), and (D) brown–black pigment deposition (hematoxylin–eosin, 10x magnification).

The autopsy demonstrated no significant external findings. Gross examination of the esophagus showed circumferential black mucosal discoloration involving the distal esophagus with sharp demarcation at the GEJ (Figure 2A). Histological sections revealed esophageal mucosal necrosis (Figure 2B) associated with transmural neutrophilic infiltrate (Figure 2C) and brown–black pigment deposition (Figure 2D). Rare fibrin thrombi were identified in the esophageal microvasculature. No significant vascular congestion or microvascular occlusion was seen. Additionally, the autopsy revealed acute tubular necrosis in the right kidney, cardiomegaly with myocyte hypertrophy and interstitial fibrosis, and diffuse alveolar damage of the lungs, predominantly involving the bilateral lower lobes with no evidence of pulmonary thromboembolism.

The cause of death was determined to be probable perioperative hypoxic–ischemic injury resulting in multiorgan failure following radical nephrectomy and IVC thrombectomy for advanced RCC. A specific underlying precipitating event could not be established despite complete autopsy examination.

## 3. Discussion

AEN, or “black esophagus,” was first described by Goldenberg et al. in 1990 [9], when the authors described a patient with black esophagus on endoscopy, resulting in a long stricture and eventually requiring esophagectomy. This entity was investigated in more detail by Gurvits et al. [1] many years later through an extensive literature review. This led to coining the term “Gurvits syndrome” as an additional terminology to describe AEN. The exact pathogenesis of AEN is unknown and it is thought to be due to a combination of ischemic compromise of the esophageal mucosa, impaired mucosal defenses, and backflow of gastric contents into the distal esophagus [6]. The distal esophagus is likely more affected by AEN as it is considered a “watershed region” that is more prone to ischemic injury, unlike the more vascularized proximal esophagus [8].

The most serious complication of AEN is esophageal perforation, which can occur in severe cases due to full‐thickness necrosis of the esophageal wall. Esophageal perforation is reported in less than 7% of AEN cases and should be suspected in rapidly decompensating patients [6]. Clinically, esophageal perforation may present with mediastinitis, empyema, and sepsis. Although imaging studies are generally not required to make the diagnosis, x‐ray or CT scan might be used to exclude esophageal perforation [8]. Early recognition of perforation, administration of intravenous antibiotics, and surgical intervention may improve patients′ survival. Other possible complications of AEN include esophageal stenosis or stricture formation and death [6].

Esophageal biopsy or cytology brushings of the affected esophageal mucosa can be supportive but are not required to make the diagnosis of AEN. Samples should be sent for microbiological analysis to rule out bacterial, viral, and fungal superimposed infections, as sepsis is a serious and well‐known complication of this condition [10]. Histologically, AEN shows loss of the normal esophageal squamous epithelium and replacement with abundant necrotic debris. The necrotic process could be limited to the mucosa or possibly extend into the submucosa and the muscularis propria leading to full‐thickness necrosis of the esophageal wall. Additional described histological findings include prominent neutrophilic infiltrate, fibrin thrombi, and deranged muscle fibers [1]. Viral inclusions may be seen, especially in immunocompromised patients.

The overall prognosis of AEN largely depends on the underlying medical conditions and the presence of complications. The role of AEN in patients′ death remains unclear given the rarity of the condition, the unknown exact pathogenesis, and the fact that many patients have one or more coexisting medical conditions that can lead to death.

The differential diagnosis of AEN includes other conditions that can lead to black discoloration of the esophageal mucosa, including malignant melanoma, acanthosis nigricans, ingestion of citric acid or sodium nitrite, coal dust deposition, and pseudomelanosis.

Treatment of AEN typically includes supportive measures with intravenous fluids, antibiotics, acid‐reducing agents, and blood transfusions. Surgical intervention for resection of the necrotic tissue and esophageal reconstruction may be required in severe cases [8].

The prevalence of malignancy in AEN ranges from 10%–29% in different studies [1, 2, 7, 11, 12]. AEN has been previously reported in association with various malignancies. However, to our knowledge, our patient is the first case report of AEN in the setting of RCC.

The potential risk factors for AEN in our patient include intraoperative hypotension and blood loss and postoperative stress and hypoperfusion, leading to esophageal mucosal ischemic injury. Additionally, the presence of advanced malignancy with IVC tumor thrombosis, necessitating IVC thrombectomy and vascular reconstruction indicates a hypercoagulable state that could have augmented the ischemic injury in this debilitated patient with poor clinical condition. Nevertheless, only focal fibrin thrombi with no significant vascular congestion or microvascular occlusion were identified on histopathological examination, suggesting that hypercoagulability played a minor role in the development of AEN in this patient.

Although AEN was unlikely to represent the primary cause of death, it may have contributed to the patient′s terminal deterioration through upper GI bleeding, additional blood loss, impaired oral intake, and amplification of the systemic inflammatory response associated with extensive esophageal necrosis. Furthermore, AEN serves as a marker of severe systemic hypoperfusion and critical illness, both of which were supported by the concurrent findings of diffuse alveolar damage and acute tubular necrosis at autopsy. Therefore, AEN in this case likely reflected both the severity of the underlying ischemic insult and a potential contributor to the patient′s overall clinical decline.

## 4. Conclusion

AEN is a rare severe condition with high morbidity and mortality. Clinicians should maintain a high index of suspicion in critically ill, older patients with multiple comorbidities presenting with GI bleeding or hemodynamic compromise. Early recognition and supportive management are essential to improve outcomes. AEN generally carries a poor prognosis with nearly one‐third of patients succumbing to the underlying critical illness. Treatment should include supportive measures and correcting the underlying medical conditions.

## Funding

No funding was received for this manuscript.

## Consent

No written consent has been obtained from the patient as there is no patient identifiable data included in this case report.

## Conflicts of Interest

The authors declare no conflicts of interest.

## Data Availability

The data that support the findings of this study are available from the corresponding author upon reasonable request.
